# YouTube as a source of information during the Covid-19 pandemic: a content analysis of YouTube videos published during January to March 2020

**DOI:** 10.1186/s12911-021-01613-8

**Published:** 2021-08-30

**Authors:** Lambodara Parabhoi, Ramani Ranjan Sahu, Rebecca Susan Dewey, Manoj Kumar Verma, Arbind Kumar Seth, Damodar Parabhoi

**Affiliations:** 1 Indian Institute of Advanced Study, Shimla, India; 2 Indian Institute of Technology, Kharagpur, India; 3 Sir Peter Mansfield Imaging Centre, The University of Nottingham, Nottingham, UK; 4 Department of Library & Information Science, Mizoram University, Aizawl, India; 5 Kendriya Vidyalaya, Nellore, India; 6 Department of Library and Information Science, Central University of Himachal Pradesh Dharamshala, Uparli Barol, India

**Keywords:** Coronavirus, COVID-19, YouTube, Video analysis, Content analysis

## Abstract

**Background:**

Institutions, government departments, and healthcare professionals engage in social media because it facilitates reaching a large number of people simultaneously. YouTube provides a platform whereby anyone can upload videos and gain feedback on their content from other users. Many YouTube videos are related to health and science, and many people search YouTube for health-related information. YouTube has been acknowledged as a key public information source in recent crises caused by Zika, H1N1, swine flu, and most recently, COVID-19.

**Methods:**

YouTube videos were collected from the YouTube Application Programming Interface (API) using the search terms COVID-19, coronavirus, COVID19, and corona. The search was conducted on April 4 and 5, 2020. The initial investigation found a total of 1084 videos. The second step involved identifying and verifying the videos for their relationship to COVID-19 information and excluding videos that did not relate to COVID-19 or were in a language other than English and Hindi.

**Results:**

An analysis of YouTube videos covering COVID-19, uploaded in early 2020, in English and Hindi. The sample comprised 349 videos (n = 334 English). Videos were characterized by contributor, duration, content, and reception (views/likes/dislikes/comments). The majority contained general information, with only 4.01% focusing on symptoms and 11.17% on treatment and outcomes. Further, the majority (n = 229) were short videos of under 10 min duration. Videos provided by government and health care professionals comprised 6.87% and 5.74% % of the sample, respectively. News channels uploaded 71.63% of videos.

**Conclusions:**

YouTube may provide a significant resource for disseminating of information on public health issues like outbreaks of viral infections and should be utilized by healthcare agencies for this purpose. However, there is currently no way to determine whether a video has been produced or verified by authorized healthcare professionals. This limitation needs to be addressed so that the vital distribution services offered by platforms like YouTube can be fully utilized for increasing public understanding of healthcare science, particularly during a crisis such as a pandemic.

## Background of the study

The global COVID-19 pandemic was caused by the spread of the coronavirus SARS-CoV-2. It was declared a pandemic by the World Health Organization (WHO) in March 2020. More recently, there has been extensive research conducted on the coronavirus that caused the pandemic. Vaccination plays an important role in preventing the spread of such viruses, but vaccines had not yet been made globally available at the time of writing.

The next line of prevention of the spread of the virus is the notion of maintaining social distancing [1, 2]. In recent years, the participation of different organizations, institutions, individuals and government departments in social media platforms such as YouTube has increased because it is one of the easiest methods to reach people. Not only do musicians, artists, and actors reach out to people through this platform but also many healthcare professionals, activists, and volunteers have become widely engaged in YouTube [3].

Participation in social media is highly gratifying, as the option of commenting on other people’s content makes the user more engaged in the content itself. The user can convey their feelings through commenting on a YouTube video, and further, commenting attracts a greater number of viewers to the video. Through these comments, the content creator can obtain feedback on their content. Commenting on a YouTube video is not just expressing a feeling but rather forms a platform for discussing the content and subject matter [4]. In recent years, YouTube videos have increased in popularity, and provided vital information sources for social sciences research [5]. There are a large number of YouTube videos related to the topic of health and science. YouTube is a major contributor to digital society, and many people seem to be searching online platforms as sources of health-related information [6].

YouTube is a key component of social media and has a great frequent users [7]. Further, with the increased availability of high-speed internet, YouTube has become very popular among the masses as an instant source of news coverage, analysis and explanation. Again, linked to the rise in internet speed, it is possible and popular to broadcast live video through YouTube. Search topics rise and fall over time, but healthcare has become a very popular search topic. It has been reported that approximately 8 out of 10 people have used YouTube to search for information relating to health [8]. The vast majority of the population now engage with at least one social media platform, and YouTube is an example of a platform where anyone can upload their content. An advantage of the way YouTube functions is that the user can subsequently share their video to other platforms through using the YouTube URL rather than uploading the entire video each time. This is a very powerful mechanism for video-sharing and has ensured the popularity of the website.

YouTube has emerged as a powerful platform for those who seek information because it provides a free online video streaming service, with the facility to download, view, upload and comment on posted videos. YouTube is now the third most popular social media site on the internet after Google and Facebook [9].

Conversely, YouTube has many limitations, including no curation of content (i.e. anyone can post a video, and it will not be removed as long as it does not breach the inappropriate content or copyright policies [10], and active monetization of content to create profit. The latter creates a bias where monetized content may be promoted more aggressively in order to increase advertising revenue and channel memberships or subscriptions. However, to address this, YouTube has limited monetizing some content that does not meet its Community Guidelines, including medical misinformation [11]. In short, content on YouTube has no requirement to be presented in a balanced or informative way.

## Objectives

1. The study seeks to analyze the increase in the prevalence of YouTube videos relating to coronavirus or COVID-19.
2. To identify the top contributors of YouTube videos relating to coronavirus or COVID-19.
3. To identify the characteristics of YouTube videos relating to coronavirus or COVID-19.
4. To determine the typical duration of a YouTube video relating to coronavirus or COVID-19.
5. To identify the most-viewed videos on YouTube relating to coronavirus or COVID-19.

## Reviews of related work

In present days YouTube has emerged as a unique source of information in the healthcare system. There has been a large amount of research conducted on YouTube videos and the content analysis of these videos [4, 7, 12, 13]. For instance, one report [12] stated that Videos on the internet, particularly on YouTube, are popular sources of public health information, despite the fact that they are often unverified. The study critically evaluated YouTube videos about the Zika virus made available during the recent Zika epidemic. A total of 101 videos were retrieved from YouTube using the search term Zika virus. The quality and reliability of these videos were evaluated using standardized tools. Videos from trusted sources like universities and health organizations were very rare. There is an urgent need for curation and authentication of health information in online video platforms like YouTube. They discuss the means to harness these platforms as useful sources of information and highlight measures that can be taken to curb the dissemination of misinformation during public health emergencies. A recent study [13] reviewed popular YouTube science video channels for evidence of attractiveness to a female audience. They investigated the influence of factors such as the gender of the presenter and the sentiment of the commenters towards males and females. Their sample was 50 YouTube science channels with a combined view count of nearly ten billion. These factors were cross-referenced with the commenter gender as a proxy for audience gender. The ratio of male to female commenters varies between 1:1 and 39:1, but the low proportions of females seem to be more related to the topic or presentation style than to the gender of the presenter or the attitudes of the commenters. Sexist behavior in YouTube commenting needs to be addressed and reduced, but the data suggests that the gender balance in the presenters of online science should not be the primary concern for channel owners. A further study [14] examined 142 YouTube videos that contained information related to the H1N1 influenza pandemic, with the aim of examining the effectiveness of YouTube as an information source during the initial phase of the outbreak. Other studies report the popularity of keyword searches of swine flu, H1N1 influenza, and influenza. It has been reported [15] that YouTube is a growing source of information about CPR, with varying degrees of quality. The authors searched YouTube using the terms CPR, cardiopulmonary resuscitation, BLS and basic life support and classified videos by upload source, content, structure, characteristics of presenters, etc. The portrayal of human papillomavirus (HPV) vaccination in video clips and viewer-posted comments available on YouTube has also been analyzed [3]. Authors used the search terms Gardasil, cervical cancer vaccination, and HPV vaccination to identify their sample, finding a total of 146 unique YouTube videos. YouTube has also been used as a resource for providing information about West Nile Virus infection, with a previous study [16] aiming to identify and evaluate YouTube as a resource for this information to the general public. Based on the information contained in the videos, they were classified as either useful, misleading, or as news updates. The authors also noted the total number of viewers, number of days since the video was uploaded, video duration, and source. A total of 106 videos were included in the study, with 79.24% having useful information about West Nile Virus.

YouTube may be a significant resource for disseminating information on public health issues like outbreaks of viral infections and should be utilized by healthcare agencies for this purpose. However, the lack of ability to determine whether a video has been produced or verified by authorized healthcare professionals is a limitation of the platform. It would be ideal if there were a process by which the content of these videos could be authorized before being made available for viewing by the community.

Some very recent studies look at the behaviors of social media users during the COVID-19 pandemic. For instance, a study of the social media platform Twitter comprised the analysis of 3,038,026 tweets in English that related to COVID-19 [17]. The study focused on the gender differences of those interacting on social media in this way. The authors concluded that females were more likely to tweet about the virus in the context of family, social distancing, and healthcare, whereas males were more likely to tweet about sports cancellations, the global spread of the virus, and political reactions. This understanding of social media usage can help policymakers inform public information announcements and understand the spread of the virus. Similarly, thematic analysis was used to investigate disability-related tweets and retweets [18]. 59 tweets posted between March 10 and April 4, 2020, were retweeted a quarter of a million times in total. By analyzing users’ information on Twitter, patterns of behavior can be observed that are informative about the level of public understanding or unrest in relation to a topic. In this instance on Twitter, it was concluded that it is unhelpful for people in less vulnerable categories to be told that their disease is less relevant because their actions can impact others through social spreading. The authors suggest that issues involving disabilities cannot be taken lightly and recommend that policymakers should carefully assess the situation to improve the delivery of healthcare services.

A significant study comparing the available literature accessed in online searches[19] reported a rapid increase in the volume of research accessible through Google Scholar and Dimensions, but not through Scopus, the Web of Science or PubMed. The findings of the study emphasize that researchers conducting literature searches with a wide scope should start with Google Scholar or Dimensions. Tweet counts and Mendeley reader counts can be used as indicators of likely significance. A recent study [20] further explores the use of Twitter during COVID-19. The authors conducted a thematic analysis of the 87 most retweeted English-language tweets (a total of 14 million retweets) that mentioned COVID-19 between March 10 and 29, 2020. They identified the main themes to be; lockdown life, attitudes towards social restrictions, politics, safety messages, people with COVID-19, support for key workers, work, and COVID-19 facts/updates. Twitter played a very important role in the dissemination of correct information and helped to increase confidence in the administration.

## Methods

YouTube hosts videos containing information related to various subjects and disciplines, including health science. YouTube videos were collected from the YouTube Application Programming Interface (API) using the search terms COVID-19, coronavirus, COVID19, and corona. The search was conducted on April 4 and 5, 2020. The initial search found a total of 1084 videos.

The second step involved identifying and verifying the videos for their relationship to COVID-19 information and excluding videos that did not relate to COVID-19 or were in a language other than English and Hindi. This stage involved removing 735 videos that were deemed irrelevant to the study due to being in a language other than English and Hindi or not being related to COVID-19). This reduced the number of videos to 349, of which 334 videos were in English and 15 in Hindi. Videos were categorized by the contributor (i.e. government, health, individual, news channel, non-profit organization), duration (minutes), type of content (i.e. death report, clinical symptoms, treatment/outcomes, general information, prevalence/precaution, lockdown), and reception (i.e. likes, dislikes, comments). The classifications of content types are as follows:

Death Report: videos that reported numbers of deaths related to COVID-19 worldwide on a daily basis starting in December 2019.

Clinical Symptoms: videos showing clinical symptoms of COVID-19 (high fever, cough, difficulty breathing, etc.) in order to increase public awareness.

Treatment/outcomes: videos describing or stating no specific treatment or vaccine for COVID-19 to date. Also, videos showing the treatment being provided by doctors, and the recovery rates from the virus, show the government recommendations to avoid community spread of the virus (washing hands, wearing face coverings or masks, avoiding direct contact, social distancing etc.).

General information: videos containing general information on COVID-19, such as background about the virus, how it is spread, and how to avoid infection.

Prevalence/Precaution: videos describing the precautions (avoiding unnecessary contact, avoiding touching the mouth, nose and eyes, keeping things clean, avoiding eating raw meat, avoiding close contact with those who have flu-like symptoms, etc.) issued by governments and the WHO in order to reduce the spread of COVID-19.

Lockdown: videos showing the effect of the lockdown imposed by various governments, in different countries around the world.

The source of each uploaded video was identified from the relevant YouTube page. These sources were grouped into mutually exclusive subtypes during analysis, as follows.

List of source categories:

Government Organization/Non-profit organization (e.g. WHO, Johns Hopkins University, MedCram): an organized group of people with a particular purpose, such as a business or government department.

Healthcare Professional OR Health Professional (e.g. Doctor, Physician): a person who studies, advises on or provides preventive, curative, rehabilitative or promotional health services based on an extensive body of theoretical and factual knowledge in the diagnosis and treatment of disease and other health problems.

Individual: an individual person who is not a healthcare professional.

News Channel (e.g. The New York Times, ABC News, DW News): a media outlet or organization.

Video duration categories:

10 minutes or shorter

Between 11 and 20 min.

Between 21 and 30 min.

Between 31 and 40 min.

Between 41 and 50 min.

Between 51 and 60 min.

61 min or longer

**Fig. 1:**
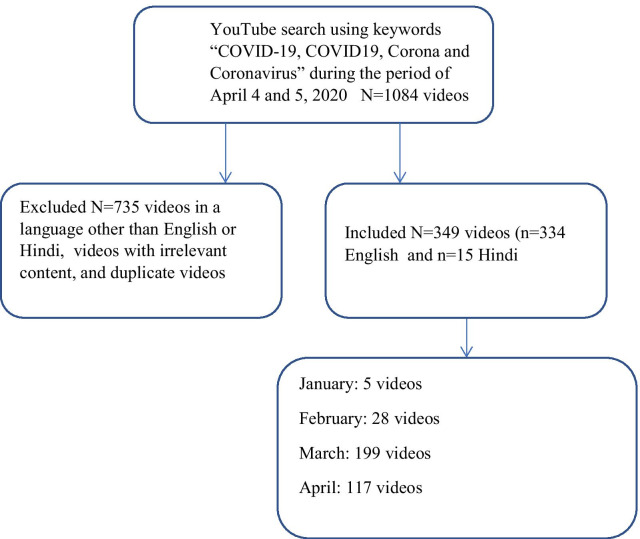
Study design

Analyses were performed in Excel to produce descriptive statistics and to tabulate the data. Finally, we characterized the top ten videos according to the number of views they received, regarding their source, duration and reception [6, 15] (Fig. 1).

## Results

1. **Growth of YouTube videos**
  Table 1 gives a broad overview of the statistics of published YouTube videos about COVID-19, by month, together with the numbers of views, likes, dislikes, and comments. Based on the published videos, the above data shows the highest number of videos were released in March, i.e., 199 out of 349 for the four-month period. Videos posted in March also received the highest numbers of views (504,729,941), likes (8,494,140), dislikes (475,843), and comments (1,192,601).
2. **Distributions of videos by language**
  Table 2 shows the language of videos published during the 4 month period. 334 videos were posted in English and 15 videos in Hindi. The 334 videos published in English attracted 824,429,849 views, 10,290,390 likes, 628,832 dislikes and 1,567,533 comments. Videos posted in the English language attracted significantly more viewers than videos posted in Hindi.
3. **Characteristics of YouTube videos on COVID-19**
  In Table 3, videos were classified as per their COVID-19 information content, regarding symptoms, death reports, general information, lockdown, precautions, and treatment. Videos uploaded by a variety of news agencies, governments, and hospital sectors with information on the current status of infections and classified as per their content. The highest number of videos published in the period (277 out of 349; 65.04%) contained general information about the disease. Out of these 277 videos, 216 videos were in English and 11 in Hindi. The majority of the videos focused on general information, with only 4.01% videos focused on clinical symptoms and 11.17% on treatment and outcomes.
4. **Distribution of videos by duration**
  Various agencies posted a total of 349 videos. Table 4 shows the numbers of videos categorized by duration. The greatest number of videos had durations of less than or equal to 10 min. These short videos received high numbers of likes, views, comments, and also dislikes. Compared to videos of other durations, these 229 short videos the were most effective, attracted the most viewers, and had the greatest impact, with 448,693,684 views, 7,333,862 likes 427,436 dislikes, and 1,129,191 comments.
5. **Top News Channels with the numbers of videos they contributed**
  The 349 videos were categorized into 139 news agencies, and news agencies were sorted according to the number of videos posted during the period. Table 5 shows the top 11 news agencies in this list, with seven or more videos each posted within the period, together with the frequency of videos published by the agency, durations of videos posted and the reception to these videos (views, likes, dislikes, comments). NBC News posted the highest number of videos, totaling 24 videos with an average duration of 16 min and 25 s. ABC News and Global News posted 17 and 16 videos, respectively, with average durations of 6 min and 5 s, and 21 min and 7 s, respectively.
6. **Distribution of videos by source**
  This section, considers the source of each video published during the study period, as shown in Table 6. Videos were categorized based on the type of organization that uploaded the video, namely whether they were a government or healthcare professional, individual, or news channel. News channels (71.63%) were the single largest source of videos, with these videos attracting the highest proportion of views, likes, dislikes, and comments. The total viewership of the 250 videos was 406,668,523 views, 4,653,425 likes, 394,296 dislikes, and 1,071,711 comments.
  The second highest contribution came from individual sources (15.75%), followed by healthcare professionals and government professionals (5.74% and 6.87%, respectively).
7. **Analysis of views, likes and dislikes**
  Table 7 shows the average and range (minimum, maximum) figures for views, likes, dislikes, and the number of comments of videos for each source.
8. **Top 10 videos on COVID-19 by numbers of views**
  The ten most highly-viewed videos published by news and media agencies during the study period are presented in Table 8. It was revealed that the video entitled “[LIVE] Coronavirus Pandemic: Real-Time Counter, World Map, News” published by Roylab Stats in English received 22,287,819 views. This was followed by the video “How wildlife trade is linked to Coronavirus” by Vox, also in English, viewed 21,469,394 times. The data also revealed that all ten of the highest-viewed videos were published in the English language. The longest video duration was 9 h, 26 min and 28 s.
9. **Summary of all videos by views, likes and dislikes**
  Table 9 gives the overall video statistics of the viewership. The videos have a combined view count of 866,430,728, with an average number of views per day of 2,482,609 (range 11,180 to 222,878,191 per day). The combined likes and dislikes received were 10,970,885 total likes (average of 31,525 per day; range 84 to 824,384 per day) and 681,967 total dislikes (average of 1960 per day; range 3 to 42,857). In total, the videos received 1,619,992 comments (average 4939 per day; range 7 to 80,747).

**Table 1:** Monthly growth of YouTube videos on subjects related to COVID-19

Month (2020)	Videos	Views	Likes	Dislike	Comments
January	05	233,785,965 (26.98%)	823,043 (7.50%)	41,417 (6.07%)	7247 (0.44%)
February	28	60,588,950 (6.99%)	876,237 (7.98%)	56,583 (8.29%)	128,915 (7.95%)
March	199	504,729,941 (58.25%)	8,494,140 (77.42%)	475,843 (69.77%)	1,192,601 (73.61%)
April	117	67,325,872 (7.77%)	777,465 (7.08%)	108,124 (15.85%)	291,229 (17.97%)
Grand total	349	866,430,728	10,970,885	681,967	1,619,992

**Table 2:** Language

Language	No. of videos	No. of views	Likes	Dislike	No. of comment
English	334	824,429,849	10,290,390	628,832	1,567,533
Hindi	15	42,000,879	680,495	53,135	52,459
Grand total	349	866,430,728	10,970,885	681,967	1,619,992

**Table 3:** Characteristics of YouTube Videos on COVID-19

Content	No. videos	%	English	Hindi	Views	Likes	Dislikes	Comments
Clinical symptoms	14	4.01	14	0	23,706,373	282,682	11,819	40,623
Death report	23	6.59	22	1	22,849,220	173,682	25,947	78,523
General information	227	65.04	216	11	681,761,669	7,951,640	546,965	1,177,715
Lockdown	18	5.15	18	0	20,363,434	178,848	20,429	69,026
Precaution	28	8.02	27	1	72,938,440	1,632,927	41,015	128,864
Treatment/outcomes	39	11.17	37	2	44,811,592	751,106	35,792	125,241
Grand total	349	100	334	15	866,430,728	10,970,885	681,967	1,619,992
Mean	58.16	SD 84.18

**Table 4:** Distribution of videos by duration

Duration	No. videos	Percentage	Total Views	Likes	Dislikes	Comments
≤ 10 min	229	65.34%	448,693,684	7,333,862	427,436	1,129,191
11–20 min	60	17.32%	94,854,090	1,709,829	126,495	293,361
21–30 min	18	5.11%	45,152,161	631,176	44,397	88,957
31–40 min	7	1.98%	10,072,791	197,807	6,339	24,255
41–50 min	5	1.42%	19,988,737	220,506	15,278	51,694
51–60 min	5	1.70%	2,907,562	13,462	1,646	3,358
≥ 61 min	25	1.70%	244,761,703	864,243	60,376	29,176
Total	349	100%	866,430,728	10,970,885	681,967	1,619,992

**Table 5:** Top news channels

News channel	No. videos	Average duration	%	Total views (average)	Likes (average)	Dislikes (average)	Comments (average)
NBC News	24	16 min 25 s	6.81	21,597,646 (899,901)	138,587 (5774)	18,971 (790)	71,991 (2999)
ABC News	17	6 min 5 s	4.8	15,799,423 (929,377)	119,268 (7015)	14,129 (831)	75,592 (4446)
Global News	16	21 min 7 s	4.54	9,907,257 (619,203)	57,698 (3606)	8675 (542)	20,991 (1399)
MSNBC	16	6 min 5 s	4.54	14,749,318 (921,832)	125,339 (7833)	35,454 (2215)	85,390 (5336)
CBS This Morning	12	6 min 8 s	3.40	15,297,506 (1,274,792)	75,972 (6331)	13,985 (1165)	42,413 (3534)
TODAY	10	12 min 3 s	2.84	14,575,708 (1,457,570)	83,661 (8361)	16,018 (1601)	56,539 (5653)
Med Cram—Medical Lectures Explained clearly	9	12 min 11 s	2.55	7,606,329 (845,147)	122,291 (13,587)	3221 (357)	19,068 (2118)
Sky News	9	4 min 44 s	2.55	25,416,031 (2,824,003)	140,146 (15,571)	8,470 (941)	31,425 (3928)
CBC News	8	25 min 8 s	2.27	6,196,428 (774,553)	61,181 (7647)	4719 (589)	17,145 (2143)
DW News	7	11 min 14 s	1.98	6,273,311 (896,187)	41,609 (5944)	3155 (450)	15,285 (2183)
Fox News	7	1 h 6 min 57 s	1.98	6,231,020 (890,145)	69,116 (9873)	7478 (1068)	28,025 (4003)
Total news agencies – 139	349

**Table 6:** Distribution of videos by source

Source	No. videos	Percentage	Total views	Likes	Dislikes	Comments
Government	24	6.87	4,28,07,052	4,85,506	2,9,228	46,189
Health Care Professional	20	5.74	7,37,23,327	17,30,868	57,990	1,33,059
Individual	55	15.75	34,32,31,826	41,01,086	2,00,453	3,69,033
News channel	250	71.64	406,668,523	4,653,425	394,296	1,071,711
Grand total	349	100	86,64,30,728	10,970,885	681,967	1,619,992

**Table 7:** Analysis of views, likes, and dislikes by the video source

Parameter	Government (range)	Average	Health (range)	Average	News channel (range)	Average	Individual (range)	Average	Non-profit organization (range)	Average
View Count	34,439,109 (47,129– 10,701,477)	2,025,829	77,914,195 (125,945–21,328,944)	3,116,567	406,668,523 (11,437–21,469,394)	1,626,674	343,231,826 (11,180–222,878,191)	8,971,216	4,177,075 (685,652–3,491,423)	2,088,537
Like	354,482 (722–98,110)	20,815	1,757,438 (1,268–824,384)	73,226	4,653,425 (84–386,767)	18,613	4,101,086 (133–732,326)	87,152	104,454 (14,401–90,053)	52,227
Dislike	26,505 (19–10,130)	1559	59,412 (128–20,991)	2475	394,296 (3–21,475)	1577	200,453 (10–42,875)	3943	1301 (304–997)	650
Comment	37,773 (248–15,993)	2360	138,024 (320–44,479)	5751	1,071,711 (7–80,747)	4639	369,033 (18–54,546)	7214	3451 (1605–1846)	1725

**Table 8:** Top 10 videos on COVID-19 by numbers of views

Title	News channel	Language	Time	View count	Like count	Dislike count	Comment count
[LIVE] Coronavirus Pandemic: Real Time Counter, World Map, News	Roylab Stats	English	9 h 28 min 28 s	222,878,191	732,326	37,452	0
How wildlife trade is linked to coronavirus	Vox	English	8 min 49 s	21,469,394	386,767	21,475	58,625
The Coronavirus Explained & What You Should Do	Kurzgesagt – In a Nutshell	English	8 min 35 s	21,328,944	824,384	9514	44,479
Journalist goes undercover at 'wet markets', where the Coronavirus started | 60 Minutes Australia	60 Minutes Australia	English	27 min 31 s	13,972,568	101,973	13,414	0
Coronavirus: Last Week Tonight with John Oliver (HBO)	Last Week Tonight	English	20 min 10 s	13,923,649	291,328	11,623	25,527
What Coronavirus Symptoms Look Like, Day By Day	Science Insider	English	5 min 19	13,543,087	182,989	5023	15,663
How To See Germs Spread (Coronavirus)	Mark Rober	English	10 min 21 s	12,443,283	454,770	6859	26,430
Coronavirus: How the deadly epidemic sparked a global emergency | Four Corners	ABC News In-depth	English	45 min 52 s	11,801,541	71,998	6736	21,659
The shocking centre of the COVID-19 crisis	Sky News	English	5 min 19 s	11,561,527	30,056	1956	0
Bill Gates makes a prediction about when coronavirus cases will peak	CNN	English	10 min 52 s	11,429,306	104,806	17,521	80,747

**Table 9:** Summary characteristics of all 349 videos by views, likes and dislikes

Characteristics	Total	Per Day	Minimum	Maximum
Total views	866,430,728	2,482,609	11,180	222,878,191
Total likes	10,970,885	31,525	84	824,384
Total dislikes	681,967	1960	3	42,857
Total comments	1,619,992	4939	7	80,747

## Conclusions and recommendations

YouTube provides a platform where any individual or organization can upload videos of any length, or broadcast live video to an audience, and currently hosts videos containing information related to various subjects and disciplines, including health science. Existing research has shown that YouTube videos have been acknowledged as a key information source for the public in recent public health crises caused by the Zika virus, H1N1 influenza, swine flu and influenza. YouTube is a commonly-consulted source of information about cardiopulmonary resuscitation, basic life support, the human papillomavirus vaccination, and the West Nile Virus infection. Social media has recently been a vital platform for the rapid dissemination of information related to the COVID-19 pandemic.

Participation in social media is highly gratifying to the users, as it offers the option of commenting on other people’s content and contributing to the viewing statistics (views, likes, dislikes). Conversely, the content creator can obtain feedback on their content. Due to this rise in popularity, YouTube videos provide a vital information source for social sciences research.

In an analysis of 349YouTube videos related to the COVID-19 pandemic, uploaded between January and April 2020, in the languages English and Hindi, the sample comprised 334videos in English and 15 in Hindi. Videos posted in March received the highest numbers of views (504,729,941), likes (8,494,140), dislikes (475,843), and comments (1,192,601). The majority of the videos focused on general information, with only 4.01% videos focused on clinical symptoms and 11.17% on treatment and outcomes. Compared to videos of other durations, short videos of between 1 and 10 min duration were the most effective, attracted the most viewers, and had the greatest impact. News channels (71.63%) were the single largest source of videos, with these videos attracting the highest proportion of views, likes, dislikes, and comments. It is conventionally believed that videos uploaded by government and healthcare professionals are the most trusted, but these two sources contributed only 6.87% and 5.74% of videos, respectively. All of the top ten videos by the number of views were posted in English. The possible explanation for this is that the English language is the internationally recognized language and that sources of information in that language have a greater capacity for impact on members of the public.

YouTube may provide a significant resource for disseminating information on public health issues like outbreaks of viral infections and should be utilized by healthcare agencies for this purpose. However, there is currently no way to determine whether a video has been produced or verified by authorized healthcare professionals. This limitation needs to be addressed so that the vital distribution services offered by platforms like YouTube can be fully utilized for increasing understanding of healthcare science, particularly during a crisis such as a pandemic.

## Data Availability

Can be available the data after approval of all authors. The corresponding author will be the responsible for providing the data. Name- Ramani Ranjan Sahu. Email Id- sahu.ramaniranjan0@gnail.com.
